# Application of magnetic motor stimulation for measuring conduction time across the lower part of the brachial plexus

**DOI:** 10.1186/1749-7221-3-7

**Published:** 2008-03-06

**Authors:** Seyed Mansoor Rayegani, Mohammad Taghi Hollisaz, Rahmatollah Hafezi, Shahriar Nassirzadeh

**Affiliations:** 1 Associate Professor of Physical Medicine and Rehabilitationn, shohada medical center, Shahid Beheshti University, M C Tehran, Iran; 2 Professor of Physical Medicine & Rehabilitation, Baghiatallah University of Medical Sciences, Tehran, Iran; 3 Assistant Professor of Physical Medicine & Rehabilitation, Baghiatallah University of Medical Sciences, Tehran, Iran; 4 Assistant Professor of Physical Medicine & Rehabilitation, Ahwaz University of Medical Sciences, Iran

## Abstract

**Objective:**

The objective of this study was to calculate central motor conduction time (CMCT) of median and ulnar nerves in normal volunteers. Conduction time across the lower part of the brachial plexus was measured by using magnetic stimulation over the motor cortex and brachial plexus and recording the evoked response in hand muscles.

**Design:**

This descriptive study was done on 112 upper limbs of healthy volunteers. Forty-six limbs belonging to men and sixty-six belonging to women were studied by magnetic stimulation of both motor cortex and brachial plexus and recording the evoked response in thenar and hypothenar muscles. Stimulation of the motor cortex gives rise to absolute latency of each nerve whereas stimulation of the brachial plexus results in peripheral conduction time. The difference between these two values was considered the central motor conduction time (CMCT).

**Results:**

In summary the result are as follows; Cortex-thenar latency = 21.4 ms (SD = 1.7), CMCT-thenar = 9.6 ms (SD = 1.9), Cortex-hypothenar latency = 21.3 ms (SD = 1.8), CMCT-hypothenar = 9.4 ms (SD = 1.8).

**Conclusion:**

These findings showed that there is no meaningful difference between two genders. CMCT calculated by this method is a little longer than that obtained by electrical stimulation that is due to the more distally placed second stimulation. We recommend magnetic stimulation as the method of choice to calculate CMCT and its use for lower brachial plexus conduction time. This method could serve as a diagnostic tool for diagnosis of lower plexus entrapment and injuries especially in early stages.

## Introduction

Magnetic motor stimulation is useful in the evaluation of a wide spectrum of nervous system disorders including multiple sclerosis, spinal cord lesions, motor neuron diseases, stroke, cervical spondylosis, intraoperative monitoring, epilepsy, pelvic floor disorders, movement disorders and some investigative conditions such as brain mapping studies [1-4].

Technical advances in this method occurred during the 1980s and this method has gained approval for clinical applications involving diagnostic and prognostic issues [5,6]. Different techniques using magnetic stimulation and normal values for each technique have not yet been studied to the same extent as conventional electrodiagnostic techniques. Cortical magnetic stimulation has remarkable advantages over electrical cortical stimulation. It is more convenient for the user, patients tolerate it much better, less time is required for magnetic stimulation and no special preparation is needed for this study.^1,2 ^Specificity of the site for magnetic stimulation is not as critical as it is for electrical stimulation [1,7].

One of the challenging topics in electrodiagnostic medicine is the diagnosis of proximal brachial plexus entrapment syndromes such as neurogenic thoracic outlet syndrome, especially in the early stages, when there is no significant axonal degeneration. At this stage there is only demyelination and/or a focal conduction block involving a short segment of plexus that can't be evaluated by routine peripheral nerve conduction studies and has no needle EMG findings. In this setting, use of Central motor conduction time (CMCT) can be a potentially useful technique to confirm the clinical diagnosis. Central motor conduction time (CMCT) is obtained when the peripheral conduction time (PCT) is subtracted from the absolute latency of cortex to target muscle conduction time. PCT is obtained by different methods including; F-wave latency, magnetic or electrical nerve root stimulation and stimulation of the brachial plexus [1,8,9]. CMCT coefficients of variation for these techniques are; 15% for cervical magnetic stimulation, 13% for F-wave latency and 11% for cervical needle electrical stimulation [10]. Facilitation and intensity of stimulation can affect all the indices of motor evoked responses including; amplitude, area and latency[1,9]. but the effects of these variants on latency of motor evoked response are far less than on area and amplitude. So the latency of motor evoked response is the most reliable index and is more frequently used for investigative purposes [1,8].

## Methods

**Figure 1:**
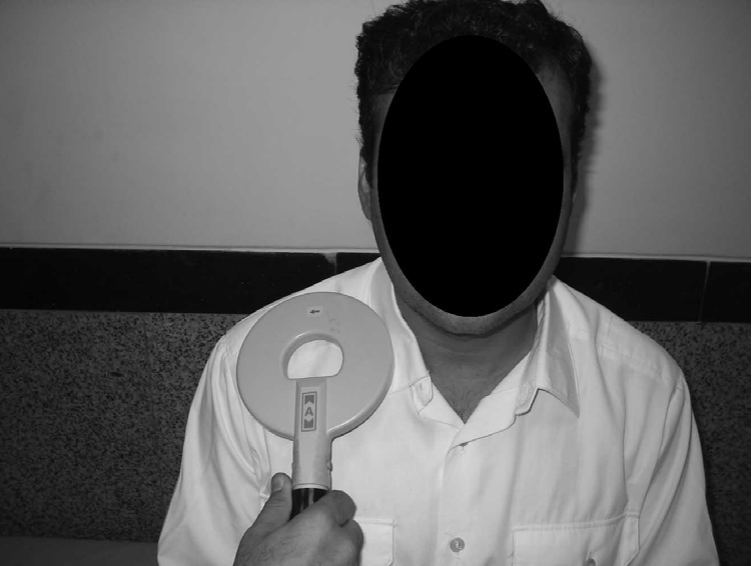
Magnetic stimulation of cortical area.

**Figure 2:**
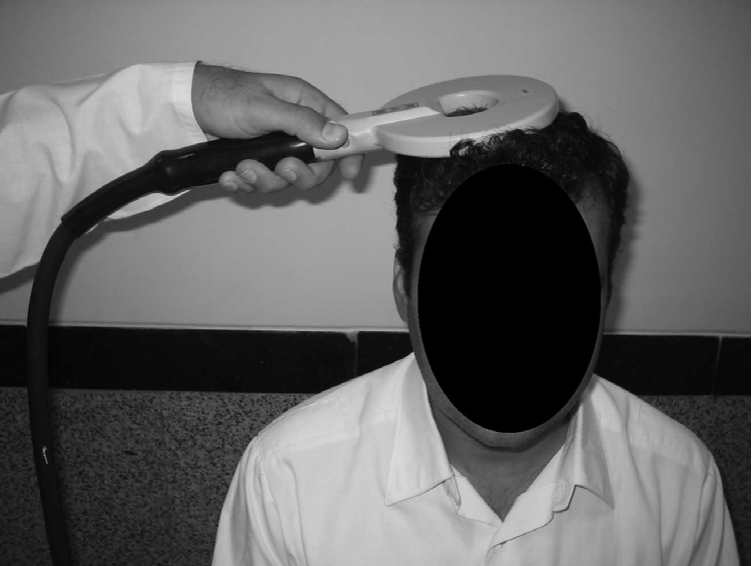
Magnetic stimulation of brachial plexus.

This study was performed in the electrodiagnostic medicine clinic of Shohada Tajrish Medical Center Tehran, Iran, between May 2006 and December 2006. Overall 112 upper limbs (66 persons) were tested, with 66 limbs belonging to healthy females and 46 belonging to healthy male volunteers. They had no history of convulsive disorders. Their neurologic clinical evaluation was normal and they had no signs of neuromuscular disorders. The medical ethics committee of Shahid Beheshti Medical University, Physical Medicine and Rehabilitation Branch approved our study. After explanation of the procedure, the volunteers signed an informed consent that was written in their native language (Persian). They were also asked if they had cardiac pacemakers, implanted metallic devices or intracranial metallic clips from neurosurgical operations. Cases having one or more of these criteria were excluded from the study. If the limb temperature was below 32°C their limbs were warmed up. All the volunteers who have undergone nerve conduction studies on upper and lower limbs and cases suspected of having neuropathies were excluded. After giving thorough explanations about the process of study the volunteers were deliberately included in the study. To obtain the absolute latencies of median and ulnar nerves, the magnetic coil stimulator was placed on the motor cortex 7 cm lateral to Cz (a line connecting both tragi together) (Figure 1) in the transverse plane and the best response was obtained from thenar and hypothenar muscles by elevating the intensity of stimulation. To obtain peripheral conduction time (PCT,) we used a second stimulation on the brachial plexus in the supraclavicular fossa by placing the magnetic coil stimulator in a plane that was parallel to the body surface (Figure 2). The recording was done on the same muscles as for cortical stimulation. The central motor conduction time (CMCT) was calculated by subtracting PCT from the absolute latency of the above mentioned nerves.

Adjustment of coil stimulator angle on the scalp and ipsilateral slight contraction of the target muscle, as the facilitation maneuvers, were used to improve the quality of response. The stimulator machine used in this study was Mag-stim 200 set on 90–100% of its maximal output (1.5 Tesla) for cortical stimulation and 70–80% of its maximal output for brachial plexus stimulation. The coil used was circular in shape with an internal diameter of 7.5 cm and its central point was used to stimulate the above mentioned targets. The recording instrument was a four channel "Toennis Neuroscreen Plus" set on: time division 5 ms, sensitivity 500–1000 μv/div. Recording electrodes were conventional bar electrodes.

## Results

Data obtained in this study was analyzed by SPSS-9 software. The mean age of males was 44.7 years (range: 24–65 yrs) and that of females was 42.0 yrs (range: 18–67 yrs). The mean for the absolute latency (cortex to muscle) of the median nerve with recording from the thenar muscles was 21.4 (SD = 1.7) ms. This value was 21.9 (SD = 1.4) ms in males and 21.0 (SD = 1.7) ms in females.

The mean for the absolute latency of the ulnar nerve with recording from the hypothenar muscles was 21.3 (SD = 1.6) ms. This value was 21.9 (SD = 1.5) ms in males and 20.9 (SD = 1.7) ms in females. The mean for the central motor conduction time (CMCT) of the median nerve with recording from the thenar muscles was 9.6 (SD = 1.9) ms. This value was 9.6 (SD = 2.0) ms in males and 9.6 (SD = 1.8) ms in females. The mean for the central motor conduction time (CMCT) of the ulnar nerve with recording from the hypothanar muscles was 9.4 (SD = 1.8) ms. This value was 9.2 (SD = 1.9) ms in males and 9.7 (SD = 1.7) ms in females (Table 1).

**Table 1:** Absolute latency and central motor conduction time (CMCT) of median and ulnar nerves in 112 upper limbs of normal volunteers

	Recording site	All patients mean(SD)	Males mean(SD)	Females mean(SD)
Absolute latency(ms)	Thenar	21.4 (1.7)	21.9 (1.4)	21.0 (1.7)
	Hypothenar	21.3 (1.6)	21.9 (1.5)	20.9 (1.7)
CMCT (ms)	Thenar	9.6 (1.9)	9.6 (2.0)	9.6 (1.8)
	Hypothenar	9.4 (1.8)	9.0 (1.9)	9.7 (1.7)

## Discussion

The number of cases entered in this study is remarkably larger than those used in similar studies. Zwarts in his study with a sample size of 36 obtained these results: latency of cortex to APB muscle = 20.6 ms (SD = 1.2) and CMCT recorded from APB = 7.4 ms (SD = 0.9) [11].

In Eisen's study with a sample size of 90, he obtained these normal values: absolute latency from cortex to thenar muscles = 20.4 ± 1.5 (16.8 – 23.8) and CMCT with thenar recording = 6.7 ± 1.2 (4.9 – 8.8) [12]. We made use of magnetic stimulation for cortical and peripheral stimulation. Our results show that there is no meaningful difference between the two genders. CMCT obtained by this method are more prolonged than values obtained when near nerve stimulation is used for PCT [8,11,12]. The reasons for this finding are: (1) PCT was obtained by brachial plexus stimulation and, (2) this was done by magnetic stimulation. These together make the PCT somewhat shorter and consequently CMCT is calculated to be longer. Some peripheral nervous system injuries such as nerve root lesions and proximal brachial plexopathies e.g. TOS, can be potentially evaluated by this method of CMCT calculation. Finally it seems that the technique for calculating CMCT as we explained in this manuscript has advantages over conventional electrodiagnostic methods, including; non-invasiveness, and convenience, taking less time from the physician., Since this method measures the proximal part of the lower brachial plexus and related ventral primary rami, it may help diagnose early stages of entrapment syndromes with mainly demyelinating and/or conduction block type of involvement. It also has its own disadvantages such as lack of specificity of stimulation site that makes its uses limited to central nervous system and long segment peripheral nervous system disorders,
